# Propofol Prevents the Growth, Migration, Invasion, and Glycolysis of Colorectal Cancer Cells by Downregulating Lactate Dehydrogenase Both In Vitro and In Vivo

**DOI:** 10.1155/2022/8317466

**Published:** 2022-04-30

**Authors:** Zhichao Wu, Han Wang, Zu-An Shi, Yalan Li

**Affiliations:** ^1^Department of Anesthesiology, Nanchong Central Hospital, The Second Clinical Medical College, North Sichuan Medical College, Nanchong, 637000 Sichuan, China; ^2^Department of Anesthesiology, The First Affiliated Hospital of Jinan University, Guangzhou, 510630 Guangdong, China

## Abstract

Colorectal cancer (CRC) is one of the most frequently diagnosed gastrointestinal malignancies worldwide and has high rates of morbidity and mortality. Propofol has been reported to have certain anticancer properties. However, the role and mechanism of propofol in CRC are not entirely clear. CRC cells were treated with propofol and/or LDH-overexpression plasmids, and a mouse xenograft model of CRC was also established and treated with propofol. Cell viability, migration, and invasion were evaluated by CCK-8, wound healing, and transwell assays; the expression of related proteins was confirmed by western blotting; indexes of the glycolytic pathway were analyzed using specialized kits; tumor growth in mice was measured; pathological tissue structure was assessed by H&E staining; and 8-OHDG expression was determined by an immunochemistry assay. Our results verified that propofol could effectively prevent the malignant behaviors of CRC cells by suppressing cell viability, migration, and invasion and accelerating apoptosis. We also discovered that propofol could attenuate the glycolytic pathway in CRC cells. Moreover, we proved that lactate dehydrogenase (LDH) was required for the inhibitory effects of propofol on the growth of CRC cells, including glycolysis in CRC cells. Furthermore, our results showed that propofol could not only significantly inhibit tumor growth and glycolysis, but also ameliorate the pathological structure of CRC tumors. The current results proved that propofol could attenuate the malignant progression of CRC by preventing LDH activity, suggesting that propofol might be an effective therapeutic agent for CRC.

## 1. Introduction

CRC is one of the most frequently diagnosed gastrointestinal malignancies worldwide [1]. According to statistics, approximately 1.36 million new cases of CRC occur each year, making the disease rank third among all malignancies worldwide. Additionally, approximately 690,000 people die from CRC each year, making it the fourth leading cause of cancer-related death worldwide [2]. Furthermore, the morbidity and mortality rates of CRC continue to rise. The current therapy for CRC is mainly surgery, supplemented by radiotherapy and chemotherapy. However, because of the occult clinical symptoms of early-stage CRC, the vast majority of patients is diagnosed with late-stage disease and cannot be cured by surgery alone. Also, the effects of chemotherapy and the prognosis for CRC patients are not ideal [3]. Data show that the 5-year survival rate of CRC patients with stage I or II disease is 90%, while that of patients with stage IV disease is only 13.1% [4]. Therefore, it is of upmost importance to gain a better understanding of the molecular mechanism of CRC progression and develop more effective therapeutic agents that can reduce the mortality rate and improve the cure rate of CRC patients.

Propofol has been used in clinical anesthesia in the form of a lipid emulsion since 1983 [5]. Currently, propofol is the most frequently used intravenous anesthetic in patients undergoing surgical removal of a tumor because of its multiple advantages, such as sedation, hypnotic effect, forgetting effect, short action time, rapid onset of action, ease of control, quick awakening time, and lack of drug accumulation [6, 7]. In addition to its anesthetic effect, propofol also has antianxiety, neuroprotective, antioxidant, immune regulatory, anti-ischemia hypoxia injury, and other effects. Cytotoxic lymphocytes play a significant role in antitumor immunity [8–10]. Research has confirmed that propofol can induce the differentiation of lymphocytes into Th1 cells and play an antitumor immune role [11, 12]. The infiltration and metastasis of cancer cells is the main cause of death among cancer patients, and 90% of patients with malignant cancer die from complications related to metastasis [13]. Literature reports indicate that propofol has significant inhibitory effects on cancer progression including the growth and metastasis of tumor cells [14–16]. Therefore, the antitumor effect of propofol is undoubtedly good news to tumor patients who require surgical treatment. However, the mechanism by which propofol prevents CRC progression remains unclear, and exploration of that mechanism is a hot area of CRC research.

In this study, we further investigated the influence of propofol on CRC progression and the mechanisms by which it exerts its influence. Additionally, we sought to identify genes that might regulate the effects of propofol in CRC.

## 2. Materials and Methods

### 2.1. Cell Culture

HCT-116 and LoVo cells were obtained from the ATCC (Manassas, VA, USA). HCT116 cells were grown in DMEM medium (Gibco, Waltham, MA, USA) supplemented with 10% fetal bovine serum (FBS, Sigma, St. Louis, MO, USA), and LoVo cells were cultured in F12K medium (Invitrogen, Waltham, MA, USA) containing 10% FBS. Both types of cells were cultured at 37°C in a 5% CO_2_ atmosphere.

### 2.2. Cell Treatment

Human LDH full-length cDNAs were amplified and then inserted into a pcDNA 3.1 vector to construct LDH-overexpression plasmids. The pcDNA 3.1 vector served as a control. HCT-116 and LoVo cells were treated either with low or high doses of propofol and were also transfected with the LDH-overexpression plasmid or vector, respectively, by using Lipofectamine 3000 (Invitrogen) for 48 h.

### 2.3. Cell Counting Kit-8 (CCK-8) Assay

Cell proliferation was determined using the CCK-8 assay. Briefly, HCT-116 and LoVo cells were seeded into the wells of 96-well plates (3000 cells/well, respectively). Next, CCK-8 reagent (Dojindo, Japan) was added to each well after 0, 24, 48, and 72 h of culture. Finally, the absorbance of each well at 450 nm was confirmed with a microplate analyzer.

### 2.4. Wound Healing Assay

The HCT-116 and LoVo cells in each group were cultured in 6-well plates (1 × 10^5^ cells/well) for 24 h. Straight lines were drawn with yellow pipette tips, and at 0 and 24 h after making the scratches, each scratch area was photographed and evaluated under an inverted microscope (Olympus IX5, Olympus America Inc.) equipped with Image-Pro Plus software. The rate of cell migration was then calculated.

### 2.5. Transwell Assay

Matrigel (20 *μ*L) was layered onto the upper membrane of a Transwell chamber at room temperature for 30 min. Treated HCT-116 and LoVo cells (200 *μ*L, 3 × 10^5^ cells/well) were inoculated into the upper compartment, and 600 *μ*L of complete medium containing 10% FBS was added to the lower compartment. After 48 h of culture, the cells in each chamber were rinsed with 1 × PBS, fixed with 5% formaldehyde for 30 min, and then stained with crystal violet for 10 min. Cells that remained in the upper compartment and had not crossed the membrane were removed with bud cotton buds, and the invaded cells were photographed under a microscope.

### 2.6. Western Blot Assay

Samples of mouse tumor tissue were ground at low temperature, and the tissue seriflux and cells in each group were added to RIPA lysate buffer. After incubation for 40 min on ice, the solution was centrifuged (13400 *g* for 15 min), and the supernatant was collected. The extracted proteins were quantified using the BCA method (Beyotime, China). Next, a 40 *μ*g aliquot of total protein from each sample was separated by 10% SDS-PAGE, and the protein bands were transferred onto PVDF membranes (Roche, Basel, Switzerland). After blocking, the membranes were incubated with primary antibodies (1: 1000) against the target proteins overnight at 4°C and then subsequently incubated with a secondary antibody (1: 2000) for 2 h. The immunostained protein bands were detected by treatment with ECL solution (Thermo Fisher Scientific, Waltham, MA, USA). The primary antibodies used (anti-caspase 3, anti-HK2, anti-PKM2, and anti-LDHA) and secondary antibody (anti-GAPDH) were purchased from Abcam (Cambridge, MA, USA).

### 2.7. ELISA Assay

Caspase 3 activity in the treated HCT-116 and LoVo cells was detected by using a Caspase 3 ELISA kit (eBioscience, San Diego, CA, USA) according to the manufacturer's instructions.

### 2.8. Determination of ATP, Lactic Acid, and Glucose Consumption

ATP, lactic acid, and glucose consumption levels were monitored using an ATP assay kit (Abnova; Taipei, KA1661), lactic acid assay kit (Sigma; MAK064-1KT), and glucose uptake colorimetric assay kit (Sigma, MAK083), respectively, according to instructions provided by the manufacturers.

### 2.9. Animals

Male BALB/c nude mice (4 weeks old) were obtained from the Animal Research Center of Jinan University and raised in the Animal Experiment Center of Jinan University. The mice were housed in a SPF room maintained at 22-24°C, 50%-60% humidity, and with a 12-h artificial light/dark cycle. The mice were fed standard feed and drank sterile water. After one week of feeding, the nude mice were subcutaneously injected with 2 × 10^6^ treated LoVo cells. Tumor size was monitored on a weekly basis, and growth curves were plotted (volume = short diameter^2^ × long diameter/2). After 28 days, the mice were sacrificed, and the tumors were collected, weighed, photographed, and used in subsequent experiments.

### 2.10. H&E Staining

The tumor tissues from nude mice was fixed with 4% paraformaldehyde, dehydrated, embedded in paraffin, and continuously cut into 4 *μ*m sections. Next, the paraffin-embedded sections of tumor were dewaxed in a xylene I, xylene II, gradient ethanol series (95%, 90%, 80%, and 70%), and distilled water. The sections were then stained with Harris hematoxylin for 5 min, immersed in 1% hydrochloric acid alcohol for 5 s, and finally immersed in 0.6% ammonia. After staining with eosin for 2 min, the sections were dehydrated with gradient ethanol and made transparent with xylene. Pathological structures in the tissue sections were examined using a microscope.

### 2.11. Immunochemistry Assay

After dewaxing and subsequent hydration, the tissue sections were immersed in 3% hydrogen peroxide and then treated with EDTA (pH = 8) for antigen recovery. Next, the sections were incubated with anti-8-OHDG antibody (Abcam) at 37°C for 1 h and then incubated with a secondary antibody at 37°C for 30 min. After washing, the sections were treated with 3, 3′-diaminobenzidine (DAB), hematoxylin, and 0.1% hydrochloric acid. After dehydration, the tissue sections were observed and photographed under a microscope.

### 2.12. Statistical Analysis

All data were analyzed using IBM SPSS Statistics for Windows, Version 21.0 (IBM Corp., Armonk, NY, USA). Measurement data are presented as a mean value ± SD. Comparisons between groups were performed using one-way analysis of variance (ANOVA), and a *P*-value <0.05 was considered to be statistically significant.

## 3. Results

### 3.1. Propofol Reduced Cell Viability, Migration, and Invasion and Upregulated Caspase 3 in CRC Cells

**Figure 1:**
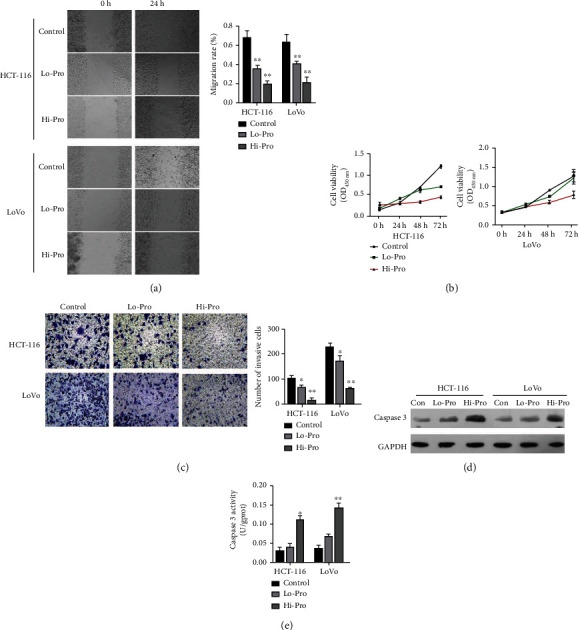
Propofol markedly repressed cell viability, migration, and invasion and upregulated caspase 3 expression in CRC cells. (a) HCT-116 and LoVo cell migration was evaluated using the wound healing assay after the cells had been treated with low or high doses of propofol. (b) CCK-8 assays were used to examine the viability of HCT-116 and LoVo cells treated with propofol. (c) Transwell assays revealed changes in the invasiveness of propofol-treated HCT-116 and LoVo cells, and the numbers of invasive cells were counted. (d) The levels of caspase 3 protein expression in HCT-116 and LoVo cells treated with propofol were detected by western blotting. (e) Caspase 3 activity in propofol-treated HCT-116 and LoVo cells was assessed using the appropriate ELISA kit.

To confirm the effects of propofol on the malignant properties of CRC cells, HCT-116 and LoVo cells were treated with two different concentrations of propofol. Subsequent wound healing assays showed that the migration rates of HCT-116 and LoVo cells were decreased in the propofol treatment groups relative to their rates in the control groups, and the inhibitory effects in the high-dose groups were more obvious than those in the low-dose groups (Figure 1(a)). CCK-8 data showed that the viability of HCT-116 and LoVo cells was markedly diminished in the propofol treatment groups (especially in the high-dose groups) when compared with the control groups (Figure 1(b)). Transwell assay results showed that the numbers of invasive cells were also significantly lower in the propofol treatment groups (especially in the high-dose groups) when compared with the control groups (Figure 1(c)). Western blot and ELISA data showed that when compared with the control cells, the levels of apoptosis-related protein (caspase 3) expression and activity were markedly higher in the low- and high-dose propofol-stimulated HCT-116 and LoVo cells and especially in the high-dose groups (Figure 1(d)). In short, these findings verified that propofol could markedly suppress the malignant progression of CRC by altering cell viability, migration, invasion, and apoptosis.

### 3.2. Propofol Inhibited Glycolysis in CRC Cells

**Figure 2:**
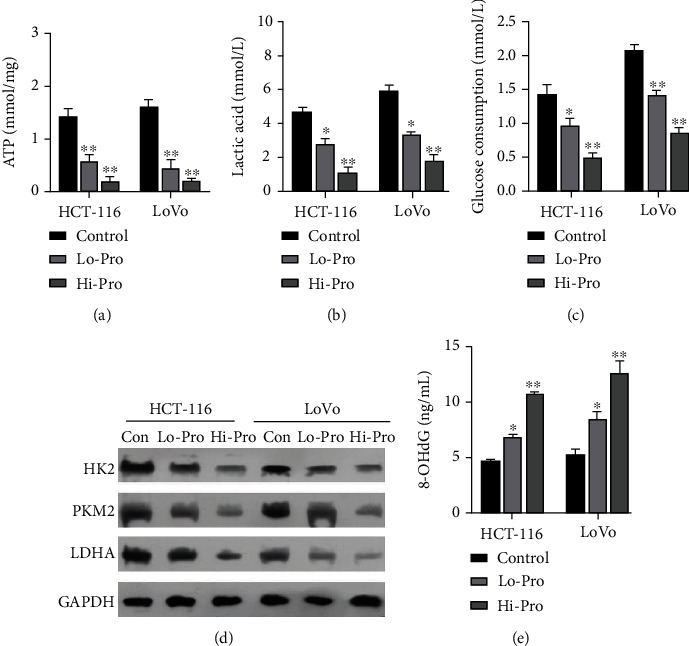
Propofol reduced glycolysis in CRC cells. HCT-116 and LoVo cells were treated with low or high doses of propofol, respectively. (a) ATP levels in the cells were determined using an ATP assay kit. (b) A lactate assay kit was used to identify changes in lactic acid content. (c) A glucose assay kit was used to analyze changes in glucose consumption. (d) Western blotting was performed to detect changes in HK2, PKM2, and LDHA protein expression. (e) An 8-OHdG assay kit was used to monitor the effect of propofol on 8-OHdG levels.

Next, we further investigated the effect of propofol on the glycolytic pathway in CRC cells. Our data revealed that the levels of ATP were dramatically decreased in the propofol treatment groups when compared with their levels in the control groups, and the effect of propofol on ATP levels in the high-dose groups was lower than in the low-dose groups (Figure 2(a)). We also discovered that lactic acid levels were marked lower in the propofol groups, and particularly in the high dose propofol groups, when compared with the control groups (Figure 2(b)). Moreover, the levels of glucose consumption were reduced in the propofol-treated HCT-116 and LoVo cells and particularly in the high-dose propofol groups (Figure 2(c)). Western blot results showed that the levels of HK2, PKM2, and LDHA protein expression in the propofol-treated HCT-116 and LoVo cells were notably decreased when compared to their levels in the control cells, and the differences were especially notable in the high-dose propofol groups (Figure 2(d)). Moreover, our results also verified that propofol, and particularly a high dose of propofol, could cause a striking increase in the levels in 8-OHdG in HCT-116 and LoVo cells (Figure 2(e)). When taken together, these data showed that propofol notably reduced the role of the glycolytic pathway in CRC cells.

### 3.3. Overexpression of LDH Weakened the Propofol-Mediated Reductions in Cell Viability, Migration, Invasion, and Induction of Apoptosis in CRC Cells

**Figure 3:**
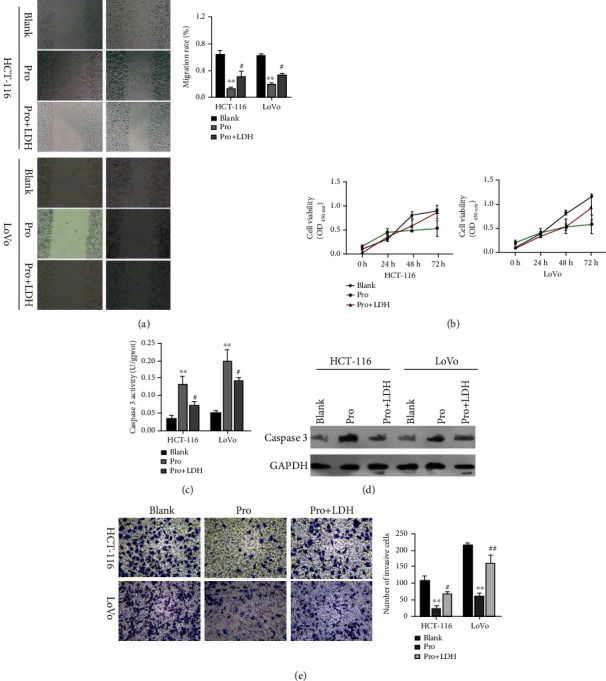
Overexpression of LDH partially attenuated the reductions in CRC cell viability, migration, and invasion mediated by propofol. Propofol or/and LDH-overexpression plasmids were used to treat HCT-116 and LoVo cells. (a) Wound healing assays were performed to detect changes in cell migration ability. (b) CCK-8 assays were performed to evaluate cell viability. (c) An ELISA kit was used to detect and quantitate caspase 3 activity. (d) Caspase 3 protein expression was analyzed by western blotting. (e) Changes in cell invasion ability were detected by Transwell assays, and the numbers of invasive cells were counted.

Next, we sought to identify genes that might regulate the action of propofol in preventing malignant progression of CRC cells. It is reported that LDH is a key enzyme in the glycolytic pathway. We speculated that LDH might play a role in CRC. To test this hypothesis, we transfected LDH-overexpression plasmids into propofol-treated HCT-116 and LoVo cells and then used the cells in several assays. Wound healing assays showed that the reductions in cell migration mediated by propofol in HCT-116 and LoVo cells could be markedly weakened by LDH overexpression (Figure 3(a)). Meanwhile, CCK-8 assays showed that cell viability, which was reduced by propofol, could be dramatically increased by LDH overexpression in HCT-116 and LoVo cells (Figure 3(b)). ELISA results showed that overexpression of LDH could markedly attenuate the enhancement of caspase 3 activity mediated by propofol in HCT-116 and LoVo cells (Figure 3(c)). Similarly, western blot results indicated that the upregulation of caspase 3 protein expression seen in propofol-treated HCT-116 and LoVo cells also could be attenuated by LDH overexpression (Figure 3(d)). Finally, transwell assay results indicated that overexpression of LDH could significantly reverse the reductions in cell invasion ability mediated by propofol in HCT-116 and LoVo cells (Figure 3(e)). When taken together, these findings indicated that propofol could attenuate the malignant progression of CRC cells by regulating LDH.

### 3.4. LDH Overexpression Reversed a Portion of Glycolysis in Propofol-Treated CRC Cells

**Figure 4:**
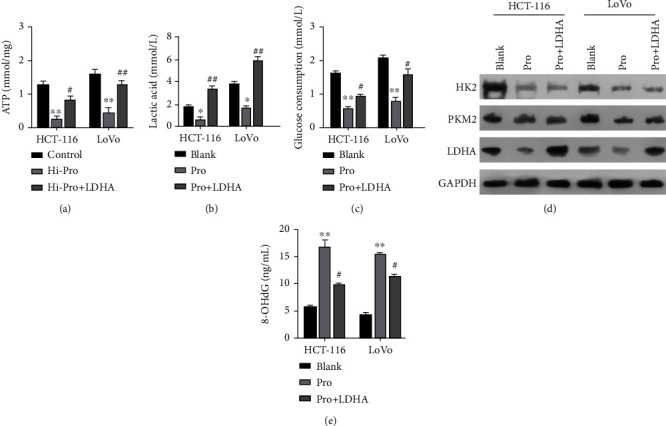
Overexpression of LDH reversed a portion of glycolysis in propofol-treated CRC cells. HCT-116 and LoVo cells were treated with propofol or/and LDH-overexpression plasmids. (a) An ATP assay kit was used to evaluate ATP levels in each group of cells. (b) A lactate assay kit was used to monitor changes in lactic acid levels. (c) Changes in glucose consumption were detected with a glucose assay kit. (d) The levels of HK2, PKM2, and LDHA proteins were detected by western blotting. (e) The levels of 8-OHdG were analyzed using an 8-OHdG assay kit.

We further investigated whether LDH was necessary for function of the glycolytic pathway mediated by propofol in CRC cells. After treatment with both propofol and LDH-overexpression plasmids, we found that the increases in ATP levels mediated by propofol in HCT-116 and LoVo cells could be markedly recovered by LDH overexpression (Figure 4(a)). Furthermore, the decreases in lactic acid levels caused by propofol treatment in HCT-116 and LoVo could be reversed by LDH overexpression (Figure 4(b)). Simultaneously, the data showed that the levels of glucose consumption, which had been reduced by propofol in HCT-116 and LoVo cells, could also be dramatically increased by LDH overexpression (Figure 4(c)). In addition, our data indicated that the decreases in HK2, PKM2, and LDHA expression mediated by propofol in HCT-116 and LoVo cells could be partially recovered by LDH overexpression (Figure 4(d)). Our data also indicated that overexpression of LDH could markedly attenuate the elevation in 8-OHdG levels mediated by propofol in HCT-116 and LoVo cells (Figure 4(e)). These data demonstrated that propofol could inhibit glycolysis in CRC cells by downregulating LDH activity.

### 3.5. Propofol Significantly Inhibited Tumor Growth and Improved Pathological Tissue Structure in a Mouse Xenograft Model of CRC

**Figure 5:**
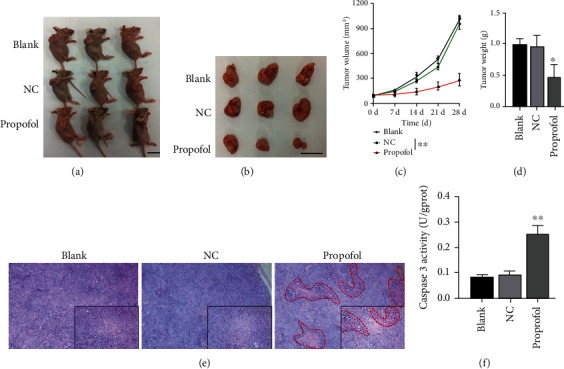
Propofol significantly inhibited tumor growth and improved pathological tissue structure in a mouse xenograft model of CRC. (a) After treatment with propofol, 2 × 10^6^ LoVo cells were subcutaneously injected into nude mice. (b) At the end of 4 weeks, the mice were sacrificed, and the subcutaneous tumors were removed for analysis. (c) Changes in tumor volume in each group of mice were measured once a week. (d) The weight of each removed tumor was measured. (e) H&E staining was performed to assess the pathological structure of tumors in each group. (f) Caspase 3 activity in each tumor was analyzed using an ELISA kit.

Our previous in vitro experiments showed that propofol could suppress the malignant progression of CRC cells by interfering with LDH. We next examined the effect of propofol in a mouse xenograft model of CRC. Tumors were formed in nude mice after a subcutaneous injection of LoVo cells, which were administrated in conjunction with propofol. Tumor growth was monitored for 28 days (Figure 5(a)). Our data showed that the tumors formed in mice in the propofol-treatment group were significantly smaller than those in the NC group (Figure 5(b)). A tumor growth curve showed that the tumor volumes in the propofol treatment group were significantly smaller than those in the NC group (Figure 5(c)). Likewise, the weights of the tumors in the propofol treatment group were markedly less than those in in the NC group (Figure 5(d)). H&E staining results revealed that the pathological structure of tumor tissues in the NC group was similar to that in the control group, and the cells in the tumors showed a disordered arrangement, an increased nucleo-cytoplasmic ratio, and a deep nucleation. When compared with NC group, the tumor cells in the propofol treatment group showed a relatively neat arrangement, a reduced nucleo-plasmic proportion, and a light nucleation (Figure 5(e)). ELISA assays revealed that caspase 3 activity was also significantly increased in the propofol treatment group when compared to that in the NC group (Figure 5(f)). When taken together, these findings indicated that propofol could markedly inhibit CRC progression in vivo.

### 3.6. Propofol Markedly Inhibited Glycolysis in the Mouse Xenograft Model of CRC

**Figure 6:**
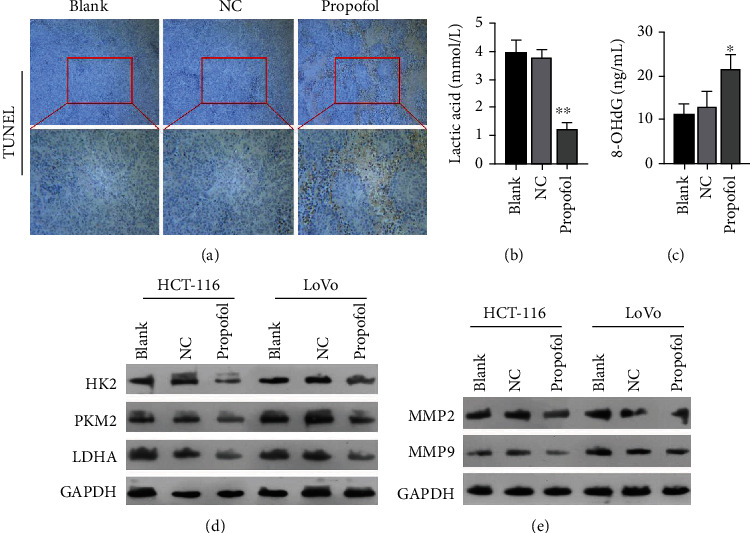
Propofol markedly inhibited glycolysis in the CRC model mice. (a) Immunochemistry assays were performed to detect 8-OHDG expression in each group of tumors. (b) An 8-OHdG assay kit was used to examine the effect of propofol on 8-OHdG levels. (c) Changes in tumor lactic acid levels were detected with a lactate assay kit. (d) The levels of HK2, PKM2, and LDHA expression in each group of tumors were examined by western blotting. (e) MMP2 and MMP9 expressions in each group of tumors were detected by western blotting.

We also explored the potential effect of propofol on the glycolysis process in the CRC model mice. As shown in Figures 6(a) and 6(b), we found that propofol treatment resulted in a marked reduction in 8-OHDG expression in the CRC tumors. Additionally, the levels of lactic acid in the propofol treatment group were dramatically greater than those in the NC group (Figure 6(c)). Furthermore, the levels of HK2, PKM2, and LDHA expression were significantly decreased in the propofol treatment group when compared with their levels in the NC group (Figure 6(d)). More importantly, western blot results showed that propofol treatment could also downregulate MMP2 and MMP9 expression in CRC tumors (Figure 6(e)). Overall, these results suggested that propofol could significantly reduce glycolysis in CRC cells as well as the metastasis of CRC cells in vivo.

## 4. Discussion

CRC is a malignant tumor of the digestive system and has high rates of morbidity and mortality [1]. Approximately 25% of CRC patients present with distant metastasis, which is the primary cause of death among CRC patients [17]. The invasion and metastasis of cancer cells is mainly regulated by tumor metastasis genes, tumor metastasis suppressor genes, tumor angiogenesis, extracellular matrix degradation, cell adhesion, the tumor microenvironment, and other factors [18]. Therefore, it is crucial to further investigate the function and mechanism of CRC metastasis and search for new drugs and biological targets for treating CSC.

In recent years, propofol has been verified to play a key role in cancer progression [14, 16]. It was reported that propofol can inhibit the activity of lipoxygenase, which is the rate-limiting enzyme for leukotriene synthesis [19]. Leukotrienes are comprised a class of vital cytokines that can accelerate the growth of human cancer cells [20, 21]. Propofol exerts an anticancer affect by altering the function of dendritic cells [22] and has been reported to prevent the proliferation and metastasis of multiple cancers. For example, propofol was shown to prevent the progress of pancreatic cancer under hypoxic conditions via ADAM8 [23] and affect the proliferation and apoptosis of cardia cancer cells via the MAPK/ERK signaling pathway [24]. Furthermore, propofol was shown to suppress the proliferation and metastasis of pancreatic cancer cells via miR-328 [25], inhibit the proliferation and cisplatin resistance of ovarian cancer cells via miR-374a [26], and inhibit the growth and migration of lung cancer cells via miR-1284 [27]. Therefore, the further research on the effect and mechanism of propofol in CRC progression might provide new ideas for developing and selecting drugs that are useful for treating CRC. In our study, we proved that propofol could markedly suppress the viability, migration, and invasion of CRC cells and induce their apoptosis. Based on the literature, we discovered that previous researchers have also found that propofol could prevent the proliferation and metastasis of CRC cells [28], which is basically consistent with our research findings. Overall, propofol has a significant inhibitory effect on CRC progression, including metastasis. However, the influence of propofol on other biological processes in CRC cells, such as glycolysis, has not been previously reported.

Tumor cells supply themselves with energy mainly via aerobic glycolysis [29]. This metabolic pathway, which differs for those in normal cells, does not require the presence or absence of oxygen and is also known as the Warburg effect [30]. Previous studies verified that the Warburg effect can result in increased glucose levels, increased glycolytic activity, and lactic acid accumulation in tumor cells [30, 31]. An acidic environment within cells can lead to instability of the extracellular matrix, which is conducive to the infiltration and metastasis of tumor cells into surrounding tissues [32]. Numerous studies have suggested that a disruption of glycolysis to reduce or eliminate the energy supply available to tumor cells may be a novel and effective strategy for cancer therapy [33, 34]. At present, the existence of aerobic glycolysis has been confirmed in multiple tumors, including bladder cancer [35], liver cancer [36], pancreatic cancer [37], oral cancer [38], breast cancer [39], prostate cancer [40], and even CRC [41]. In our study, we used glycolysis-related indicators to further show that propofol could markedly block the glycolysis process in CRC cells. Moreover, another study demonstrated that propofol could disrupt aerobic glycolysis in lung cancer [42], which further confirms the validity of our current results.

LDH, as an essential enzyme in the glycolytic pathway, is crucially important for the mutual conversion of pyruvate and lactic acid [43]. Several studies have shown that LDH levels can be abnormally elevated in a variety of solid tumors, and such elevated levels are associated with a drug resistance, a poor response to treatment, and a poor prognosis [44–46]. LDH has five isoenzymes that are based on its constituent subunits (M subunit and H subunit) [44, 47]. Among those isoenzymes, LDHA, which is composed entirely of M subunits, catalyzes the conversion of pyruvate to lactic acid [48]. Studies have also verified that an elevated LDHA level is associated with the degree of tumor differentiation, progression, and the prognosis of a cancer [49, 50]. LDHA, together with HK2 and PKM2, are the key rate-limiting enzymes in glycolysis [51]. Therefore, blocking the activities of these key rate-limiting enzymes associated with glycolysis may decrease the energy produced by tumor glycolysis and have an anticancer effect. In our study, we also proved that propofol could significantly downregulate HK2, PKM2, and LDHA expression in CRC cells. More importantly, we confirmed for the first time that overexpression of LDH could partially reverse cell viability, migration, invasion, apoptosis, and glycolysis, which were mediated by propofol in CRC cells, suggesting that propofol could attenuate CRC progression, including glycolysis, by downregulating LDH in vitro and in vivo. Research shows that LDHA overexpression is associated with malignant behavior of tumor cells. For example, the study of Sheng et al. showed that LDHA can promote the expression of MMPs in liver cancer, and the interference of LDHA can inhibit tumor metastasis [52]. However, LDHA mediates the production of lactate, which is released by the monocarboxylate transporters (MCTs), exacerbating environmental acidification and promoting environmental support for tumor metastasis [53]. In this study, we detected an increase in lactate in animal tumors, accompanied by an upregulation of MMP2/9 expression, most likely in conjunction with an increase in lactate efflux leading to increased extracellular acidification, resulting in increased cell invasive potential.

**Figure 7:**
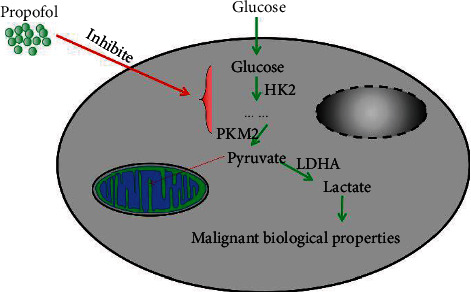
Graphic abstract.

In conclusion, our current study demonstrated that propofol can inhibit CRC progression by suppressing LDH expression, which in turn prevents glycolysis and the proliferation and metastasis of CRC cells (Figure 7). Therefore, we conclude that propofol might be a drug that is useful for treating CRC and LDH might be an effective target for CRC therapy.

## Data Availability

The data used to support the findings of this study are included within the article.
